# A new structural mode of emotional empathy: relationship of emotional comparison, ‎perspective taking, Feminine trait, emotion regulation and self-monitoring with emotional ‎empathy of students 

**Published:** 2019-08

**Authors:** Salman Abdi

**Affiliations:** ^*a*^Tabriz University of Medical Sciences, Road Traffic Injury Research Center, Tabriz, Iran.

## Abstract

**Background:**

Emotional empathy is defined as one of the basic concepts in social perception. The present ‎study aimed at presenting a new model of emotional empathy, determining structural relation of ‎perspective taking, emotional comparison, Feminine traits, self-monitoring, and emotion ‎regulation with emotional empathy of the students. ‎

**Methods:**

In this study, 503 students of Tabriz University were evaluated using Balance Emotional ‎Empathy Scale (BEES), Interpersonal Reaction Inventory (IRI), Cognitive Emotion Regulation ‎Questionnaire (CERQ), Mindfulness Attention Awareness Scale (MAAS), Bem Sex Role ‎Inventory (BSRI), revised Self-Monitoring scale (RSMS), Emotional Comparing Scale (ECS). ‎Fitness indexes of the structural model of emotional empathy were determined using ‎LISREL.8.54 software. ‎

**Results:**

Mean age of the subjects was 21.53±2.41. There was a direct structural relationship between ‎emotional empathy and feminine traits, emotional comparison, perspective taking, adjusted ‎emotion regulation, and unadjusted cognitive emotion regulation, respectively. There is an ‎indirect relationship between emotional empathy and sensitivity to the expressive behavior of ‎others (CFI=0.89, IFI=0.90, RMSEA=0.053). ‎

**Conclusions:**

According to this preliminary study, constructs of emotional comparison, sensitivity to the ‎expressive behavior of others, and emotion regulation play a significant role in emotional ‎empathy or emotional perception. The significance of social comparison was discussed in the ‎model of emotional empathy.‎

**Keywords:**

Emotional empathy, Feminine traits, Emotional comparison, Perspective taking, Self-‎monitoring, Cognitive Emotion regulation, Mindful

